# FANCD2 as a novel prognostic biomarker correlated with immune and drug therapy in Hepatitis B-related hepatocellular carcinoma

**DOI:** 10.1186/s40001-023-01411-0

**Published:** 2023-10-11

**Authors:** Xiaowei Tang, Bei Luo, Shu Huang, Jiao Jiang, Yuan Chen, Wensen Ren, Xiaomin Shi, Wei Zhang, Lei Shi, Xiaolin Zhong, Muhan Lü

**Affiliations:** 1 Department of Gastroenterology, the Affiliated Hospital of Southwest Medical University, Luzhou, China; 2 Department of Gastroenterology, the People’s Hospital of Lianshui, Huaian, China

**Keywords:** FANCD2, Hepatocellular carcinoma, Biomarker, Prognostic, Immunity

## Abstract

**Background:**

Ferroptosis is related to the immunosuppression of tumors and plays a critical role in cancer progression. Fanconi anemia complementation group D2 (FANCD2) is a vital gene that regulates ferroptosis. However, the mechanism of action of FANCD2 in Hepatitis B-related hepatocellular carcinoma (HCC) remains unknown. In this study, we investigated the prognostic significance and mechanism of action of FANCD2 in Hepatitis B-related HCC.

**Methods:**

The expression of FANCD2 in Hepatitis B-related HCC was explored using The Cancer Genome Atlas (TCGA) and validated using the Gene Expression Omnibus (GEO) database. Univariate and multivariate Cox regression analyses and Kaplan–Meier survival curves were used to analyze the relationship between FANCD2 expression and the overall survival of patients with Hepatitis B-related HCC. Protein–protein interaction networks for FANCD2 were built using the STRING website. In addition, correlations between FANCD2 expression and the dryness index, tumor mutational burden, microsatellite instability (MSI), immune pathways, genes involved in iron metabolism, and sorafenib chemotherapeutic response were analyzed.

**Results:**

Our results indicated that FANCD2 was significantly overexpressed in Hepatitis B-related HCC and demonstrated a strong predictive ability for diagnosis (Area Under Curve, 0.903) and prognosis of the disease. High FANCD2 expression was associated with poor prognosis, high-grade tumors, high expression of PDL-1, high MSI scores, and low sorafenib IC50 in Hepatitis B-related HCC. BRCA1, BRCA2, FAN1, and FANCC were vital proteins interacting with FANCD2. The expression level of FANCD2 significantly correlated with the infiltration levels of Treg cells, B cells, CD8 + T cells, CD4 + T cells, neutrophils, macrophages, myeloid dendritic cells, and NK cells in Hepatitis B-related HCC. FANCD2 was positively correlated with the tumor proliferation signature pathway, DNA repair, and cellular response to hypoxia.

**Conclusion:**

Our study indicated that FANCD2 was a potential novel biomarker and immunotherapeutic target against Hepatitis B-related HCC, which might be related to the chemotherapeutic response to sorafenib.

## Introduction

One of the biggest challenges associated with malignant tumors is hepatocellular carcinoma (HCC), the incidence and mortality of which have increased worldwide [1]. The most crucial cause of HCC is hepatitis B virus (HBV) infection [2]. According to some studies, Hepatitis B-related HCC accounts for approximately 80% of all liver cancer cases [3]. Poor prognosis is often associated with liver cancer. Liver cancer is often diagnosed at an advanced stage [4]. Immunotherapy and molecular therapy have gained momentum in recent years, improving the overall and disease-free survival of patients and disease-free survival [5]. However, individuals respond differently to immunotherapy and molecular-targeted therapies. Immune escape has been a major obstacle to HCC treatment [6].

Additionally, Hepatitis B-related HCC has a poorer prognosis [7]. Thus, identifying effective biomarkers is crucial for the early diagnosis, treatment, and prognostic evaluation of Hepatitis B-related HCC, which can help optimize healthcare resources and improve patient outcomes.

Ferroptosis, a recently discovered form of cell death, has attracted significant attention as a potential therapeutic target in carcinoma [8]. Researchers have suggested that ferroptosis plays a vital role in the development and progression of various cancers [9]. Ferroptosis has been associated with various malignancies, including ovarian, kidney, and liver cancers [10]. Ferroptosis regulates tumor progression by enhancing the efficacy of chemotherapy, immunotherapy, and molecular-targeted therapies [7, 11, 12]. Ferroptosis-related genes are therapeutic targets and prognostic biomarkers in some cancers [13, 14]. The ferroptosis-related gene Fanconi anemia complementation group D2 (FANCD2) is a suppressor of ferroptosis and plays an essential role in cell cycle progression, replication, and repair [15–17]. Previous studies have shown that FANCD2 plays a role in regulating the microenvironment of carcinomas and influencing their metastasis, proliferation, and apoptosis [18–21]. Additionally, FANCD2 is associated with the proliferation and invasion of HCC cells [22]. Thus, FANCD2 has the potential to become an attractive target for understanding and treating cancer. However, there are no relevant studies on the effects of FANCD2 on Hepatitis B-related HCC.

Therefore, this study aimed to analyze the prognostic significance and mechanism of the ferroptosis-related gene FANCD2 in Hepatitis B-related HCC to predict therapeutic response.

## Methods

### Data collection and expression

We downloaded expression profiles and clinical data from the TCGA dataset (The Cancer Genome Atlas, https://portal.gdc.nih.gov) for patients with Hepatitis B-related HCC and other cancers. Expression samples from normal humans were downloaded from GTEx (http://commonfund.nih.gov/GTEx/). R software version 4.0.3 (R Foundation for Statistical Computing, Vienna, Austria) was used to conduct statistical analyses. First, we analyzed the pan-cancer expression of FANCD2. We then studied the expression of FANCD2 in different genders, races, and tumor stages in Hepatitis B-related HCC. Furthermore, the expression of FANCD2 in Hepatitis B-related HCC was verified using the GSE121248, GSE55092, GSE19665 and GSE84402 datasets.

### Prognostic and diagnostic roles of FANCD2

The TCGA dataset was used to examine whether FANCD2 expression was related to survival time and survival status in Hepatitis B-related HCC. The survival difference between the high-expression of the FANCD2 and low-expression FANCD2 groups was evaluated using the log-rank test. Based on log-rank tests and univariate Cox proportional hazards regression, we plotted Kaplan–Meier curves, calculated p-values, and hazard ratios (HR) with 95% confidence intervals (CI). We compared predictive accuracy using time ROC (v 0.4) analysis. The relationship between FANCD2 expression and the prognosis of patients with Hepatitis B-related HCC was investigated using univariate and multivariate analyses. Based on the multivariate Cox proportional hazards analysis, a nomogram was developed to predict overall survival in the first, second, and third years. In addition, we calculated the diagnostic Area Under Curve (AUC) based on the expression data of FANCD2.

### Correlation analysis of FANCD2 and immunity

We used the TIMER database (https://cistrome.shinyapps.io/timer/) to investigate the relationship between FANCD2 expression and the abundance of infiltrating immune cells using TIMER and QUANTISEQ algorithms. We selected SIGLEC15, TIGIT, CD274, HAVCR2, PDCD1, CTLA4, LAG3, and PDCD1LG2 as immune checkpoints and analyzed the correlation between their expression and the expression of FANCD2 in Hepatitis B-related HCC. Spearman’s correlation analysis determined the correlation between FANCD2 expression, immune cells, and immune checkpoints. The potential immune checkpoint blockade (ICB) response was predicted using the tumor immune dysfunction and exclusion (TIDE) algorithm.

### Protein–protein interaction network and mutations analyses of FANCD2

We used STRING to analyze the proteins interacting with FANCD2 and constructed a protein–protein interaction (PPI) network. We downloaded and visualized the mutation data using the maftools package in the R software. The tumor mutation burden (TMB) and microsatellite instability (MSI).

### Dryness index and pathway

First, the relationship between the dryness index and FANCD2 expression was analyzed. Using Spearman correlation, we calculated mRNAsi using the one-class linear regression (OCLR) algorithm and explored the relationship between the mRNAsi score and FANCD2 expression. Based on the expression data, mRNAsi was calculated and ranged from 0 to 1. In general, the closer the index is to 1, the lower the degree of differentiation of the tumor cells and the stronger the characteristics of the tumor stem cells. Second, the correlation between FANCD2 and the pathway was investigated using Spearman’s correlation. Analysis was conducted using R software, package GSVA, with parameter method = “ssgsea.” We also investigated the correlation between FANCD2 and the genes involved in iron metabolism, including FTH1, HAMP, HSPB1, SLC40A1, STEAP3, TF, and TFRC.

### Correlation analysis of IC50 score of Sorafenib and FANCD2

We used the Genomics of Drug Sensitivity in Cancer (GDSC) database (https://www.cancerrxgene.org), the largest publicly available pharmacogenomic database, to predict therapeutic responses for each sample. The prediction process was carried out using the R package "pRRophetic." We calculated the samples' half-maximal inhibitory concentration (IC50) using the ridge regression method. Using Spearman's correlation, we then analyzed the relationship between sorafenib IC50 score and FANCD2 expression.

## Results

### The mRNA levels of FANCD2 in Hepatitis B-related HCC and pan cancer

**Fig. 1:**
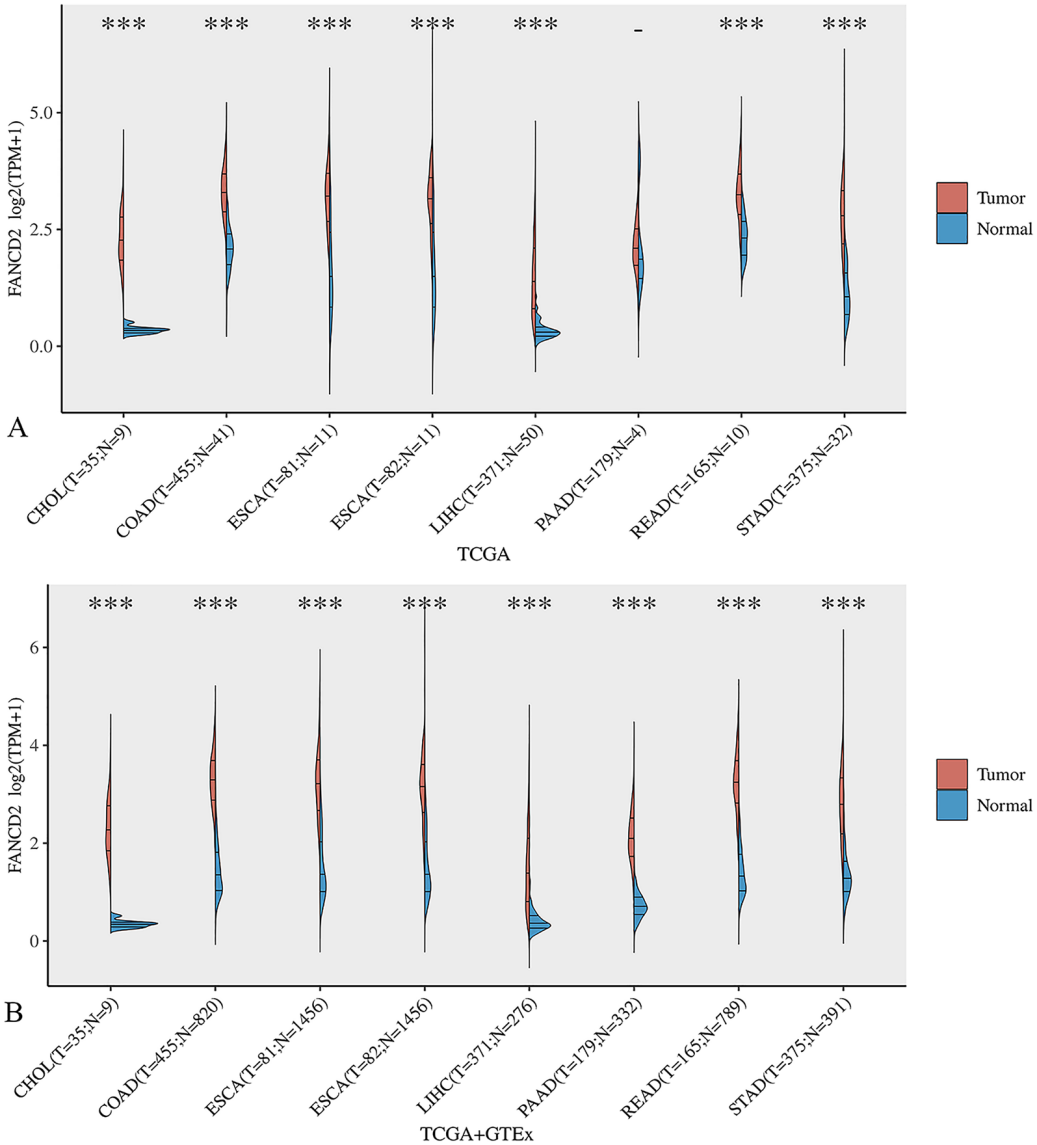
The expression of FANCD2 in different cancers. **A** The expression of FANCD2 in different cancers and adjacent normal tissues in the TCGA database. **B** The expression of FANCD2 in different cancers and normal tissues of GTEx. CHOL, cholangiocarcinoma; COAD, colon adenocarcinoma; LIHC, Liver hepatocellular carcinoma; PAAD, pancreatic adenocarcinoma; READ, rectum adenocarcinoma; STAD, stomach adenocarcinoma

**Fig. 2:**
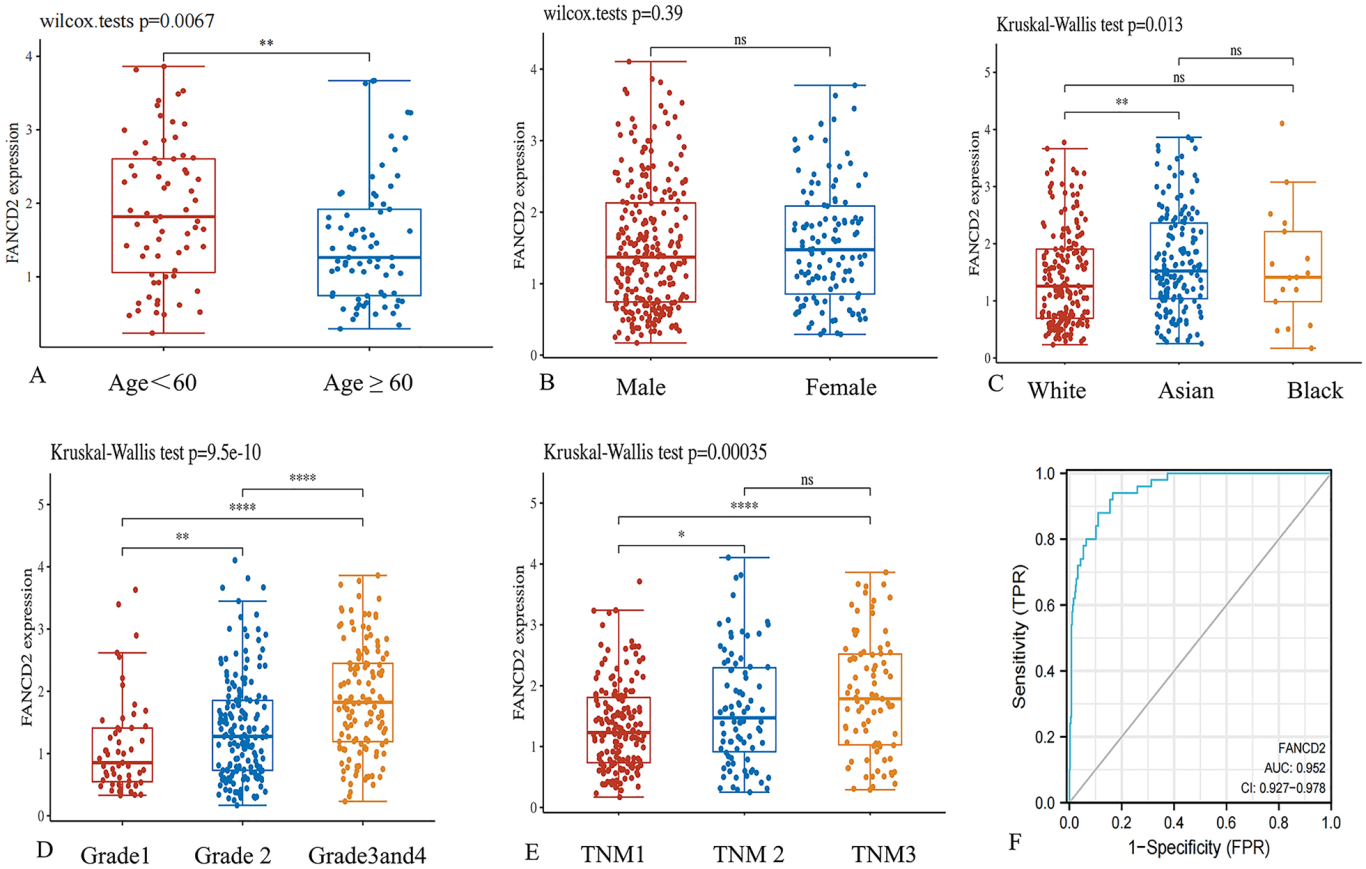
The expression of FANCD2 in Hepatitis B-related hepatocellular carcinoma in the TCGA database. The expression of FANCD between different age, gender, race, grade stages, and TNM stages (**A–E**). **F** The Area Under Curve (AUC) for FANCD2 in the diagnosis of Hepatitis B-related hepatocellular carcinoma

In the (TCGA) dataset, the expression of FANCD2 in many types of cancers, including HCC and Hepatitis B-related HCC, was higher than that in normal tissues (Fig. 1). We analyzed the correlation between FANCD2 and HCC patients in different age groups and found that people older than 60 years old were highly expressing FANCD2 compared to people younger than 60 years old (Fig. 2A). According to our analysis, there was no statistical difference in FANCD2 expression between males and females in hepatitis B-associated hepatocellular carcinoma (Fig. 2B). In Hepatitis B-related HCC, FANCD2 expression was highest in Asian patients compared to black and white patients, according to our analysis (Fig. 2C). The expression of FANCD2 was also different in different grades and TNM stages, with higher expression in grades 3, 4, and TNM 2 and 3 (Fig. 2D and E). Figure 2F shows the high diagnostic accuracy of FANCD2 in predicting Hepatitis B-related HCC (AUC, 0.903; 95% CL, 0.839–0.966). Furthermore, we verified that the expression of FANCD2 was higher in Hepatitis B-related HCC than in normal tissues in GSE121248 (*P* = 5.9e−10), GSE55092 (*P* = 0.0021), GSE19665 (*P* = 1.1e−05) and GSE84402(*P* = 0.00034).

### Higher FANCD2 mRNA expression showing poor prognosis in Hepatitis B-related HCC

**Fig. 3:**
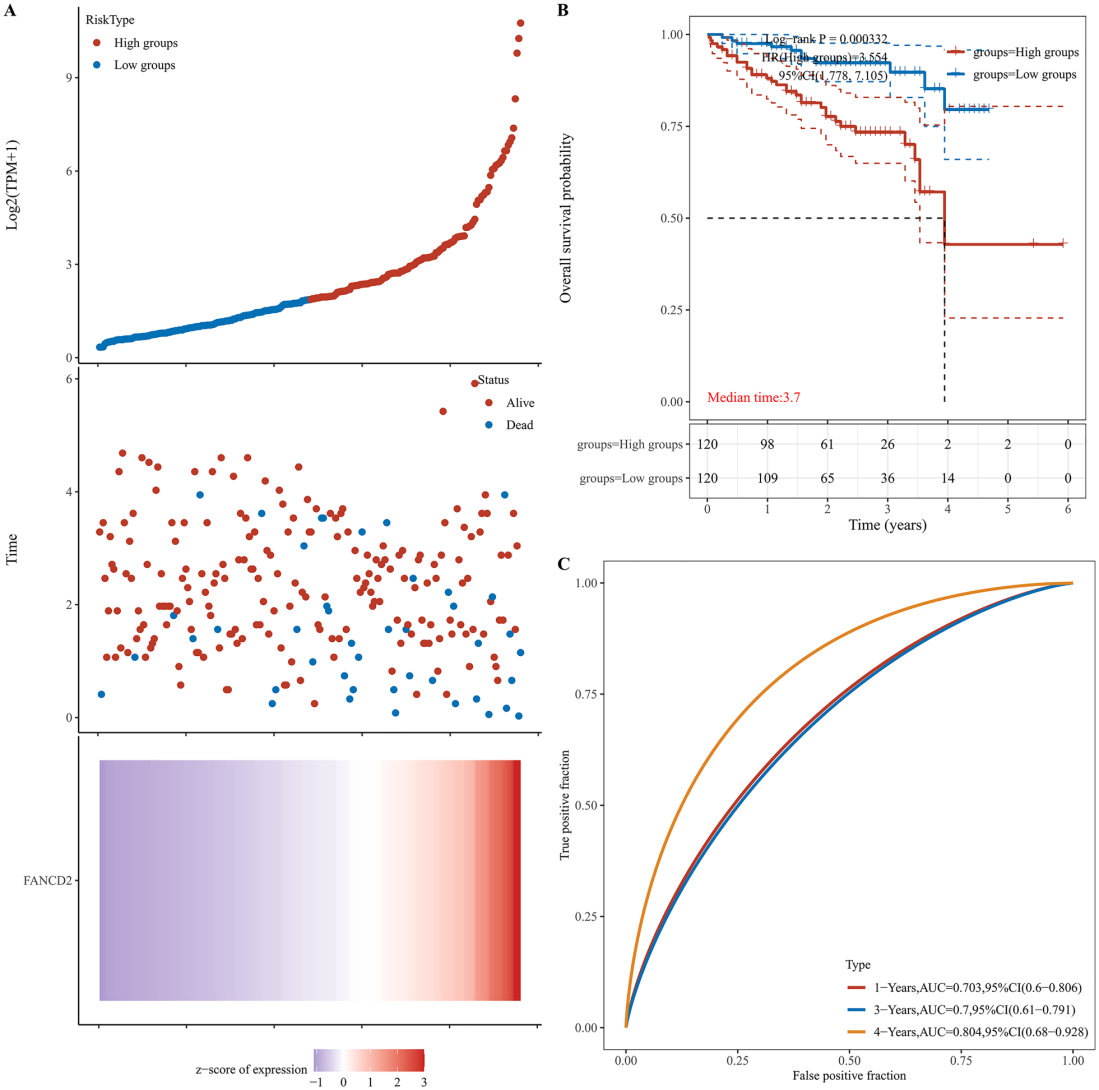
The relationship between FANCD2 expression and survival status in Hepatitis B-related hepatocellular carcinoma. **A** The relationship between FANCD2 expression and survival time and survival status. The top plot represents a scatter plot of FANCD2 expression from low to high. The middle plot represents the scatter plot distribution of survival time and survival status corresponding to FANCD2 expression in different samples. Bottom plot represents the expression heatmap of FANCD2; **B** KM survival curves of the FANCD2; **C** Receiver Operating Characteristic (ROC) curves and Area Under Curve (AUC) values for FANCD2 in the first year, third year, and fourth years

**Fig. 4:**
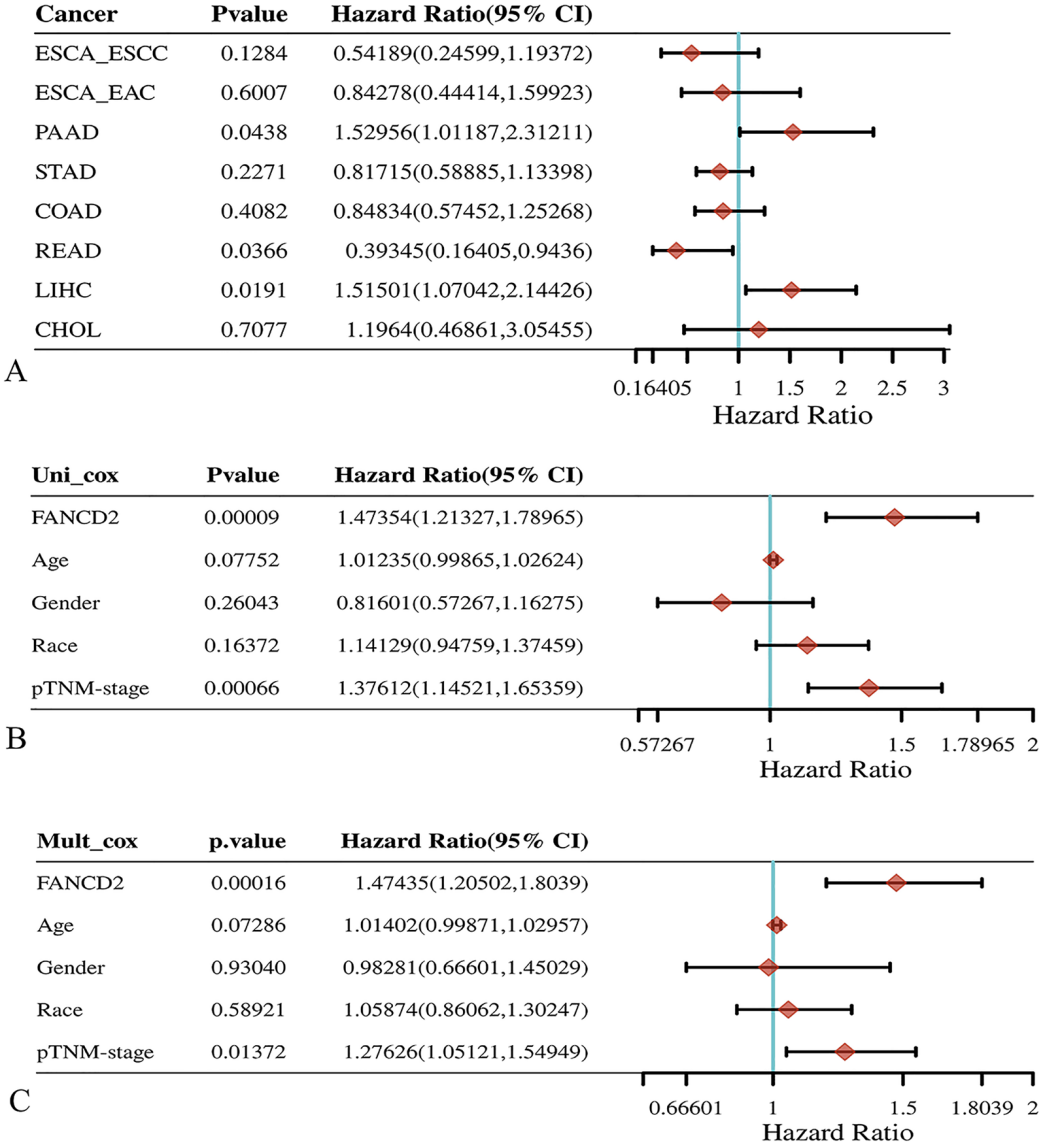
The relationship between FANCD2 and cancer prognosis. FANCD2 univariate cox analysis in different cancers (**A**) and Hepatitis B-related hepatocellular carcinoma (**B**). **C** FANCD2 and clinical characteristics multivariate cox analysis in Hepatitis B-related liver cancer

**Fig. 5:**
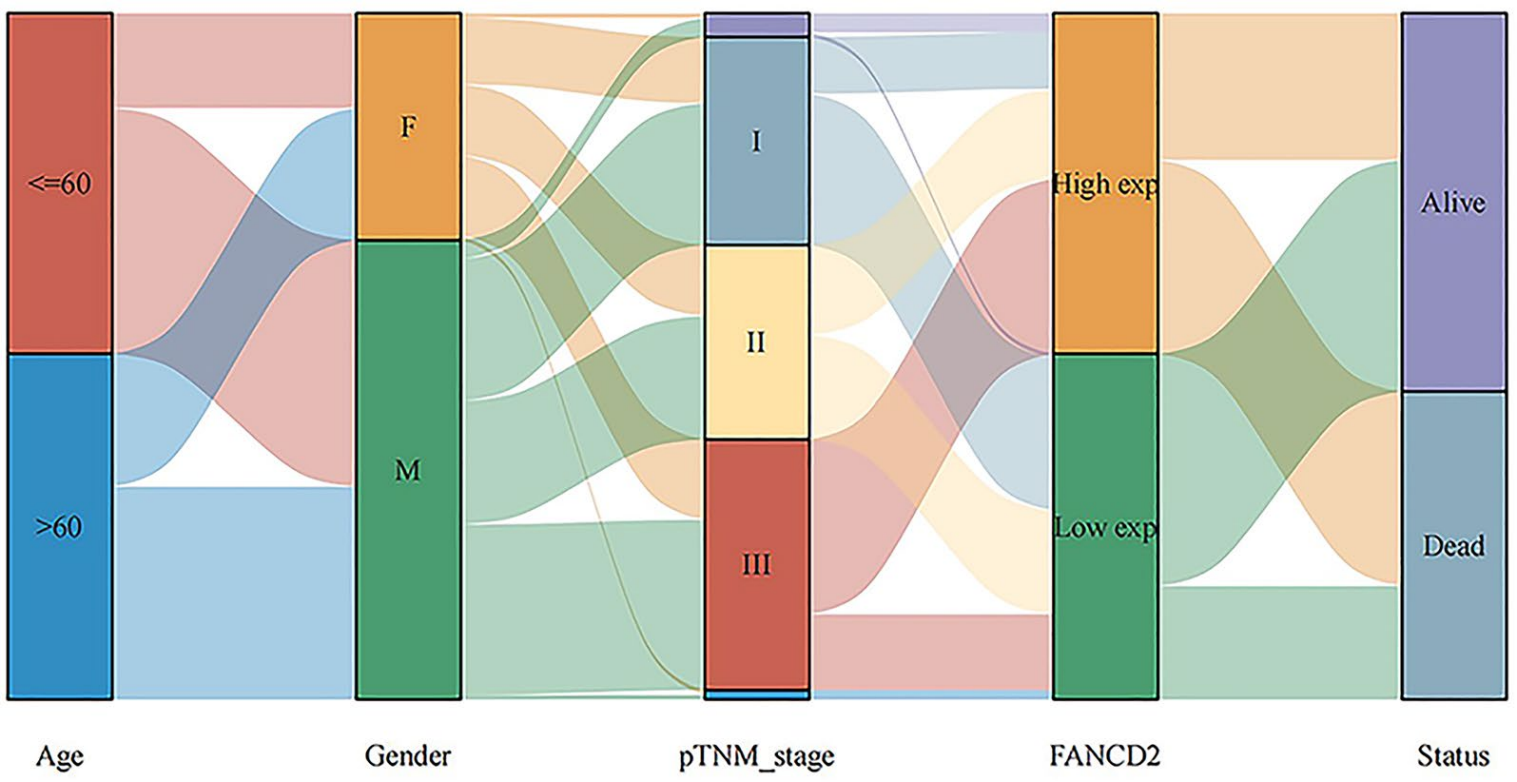
A Sankey diagram about age, gender, pTNM stage, the expression of FANCD2 and the survival status of the patients. Each column represents a variable and the lines between columns represent correlations. The first column represents age and the second column represents gender. Based on the connecting line between the first and second columns, it can be seen that male patients predominate, whether among those older than 60 or younger than 60 years of age. The third column represents TNM staging, with the majority of male and female patients in TNM stage I. The fourth column shows that the expression of FANCD2, FANCD2 was mostly under-expressed in patients with TNM stage I, while in patients with TNM stage III, FANCD2 was mostly over-expressed. The fifth column represents the survival status of the patients, patients with high FANCD2 expression went to death more often than patients with low FANCD2 expression

In Hepatitis B-related HCC, high FANCD2 expression had a poor overall survival (OS, Log-rank P, 0.000193; HR, 2.689; 95% Cl, 1.599–4.524), progression-free survival (PFS, Log-rank P, 0.000439; HR, 2.284; 95% Cl, 1.441–3.619), disease-free survival (DFS, Log-rank P, 0.00107; HR, 2.391; 95% Cl, 1.418–4.029) and disease-specific survival (DSS, Log-rank P, 0.000833; HR, 3.092; 95% Cl, 1.595–5.996) in TCGA database (Fig. 3). Figure 3C shows the 1-year prognostic AUC of the FANCD2 was 0.703 (95% Cl, 0.6–0.806), the 3-year prognostic AUC was 0.7 (95% Cl, 0.61–0.791), and the 4-year prognostic AUC was 0.804 (95% Cl, 0.668–0.928) in OS. Figure 4A shows the results of the univariate Cox analysis of FANCD2 prognosis in various cancer types. FANCD2 was also identified as a risk factor in the univariate Cox regression analysis (P < 0.0001; HR, 1.90221; 95% Cl, 1.45487–2.4871) and in the multivariate Cox regression analysis (P < 0.0001; HR, 1.82417; 95% Cl, 1.36047–2.44593) (Fig. 4B and C). Figure 5 is a Sankey diagram about age, gender, pTNM stage, the expression of FANCD2 and the survival status of the patients. Each column represents a variable and the lines between columns represent correlations. The first column represents age and the second column represents gender. Based on the connecting line between the first and second columns, it can be seen that male patients predominate, whether among those older than 60 or younger than 60 years of age. The third column represents TNM staging, with the majority of male and female patients in TNM stage I. The fourth column shows the expression of FANCD2, FANCD2 was mostly under-expressed in patients with TNM stage I, while in patients with TNM stage III, FANCD2 was mostly over-expressed. The fifth column represents the survival status of the patients, patients with high FANCD2 expression went to death more often than patients with low FANCD2 expression.

### Correlation between FANCD2 expression and immune characteristics

**Fig. 6:**
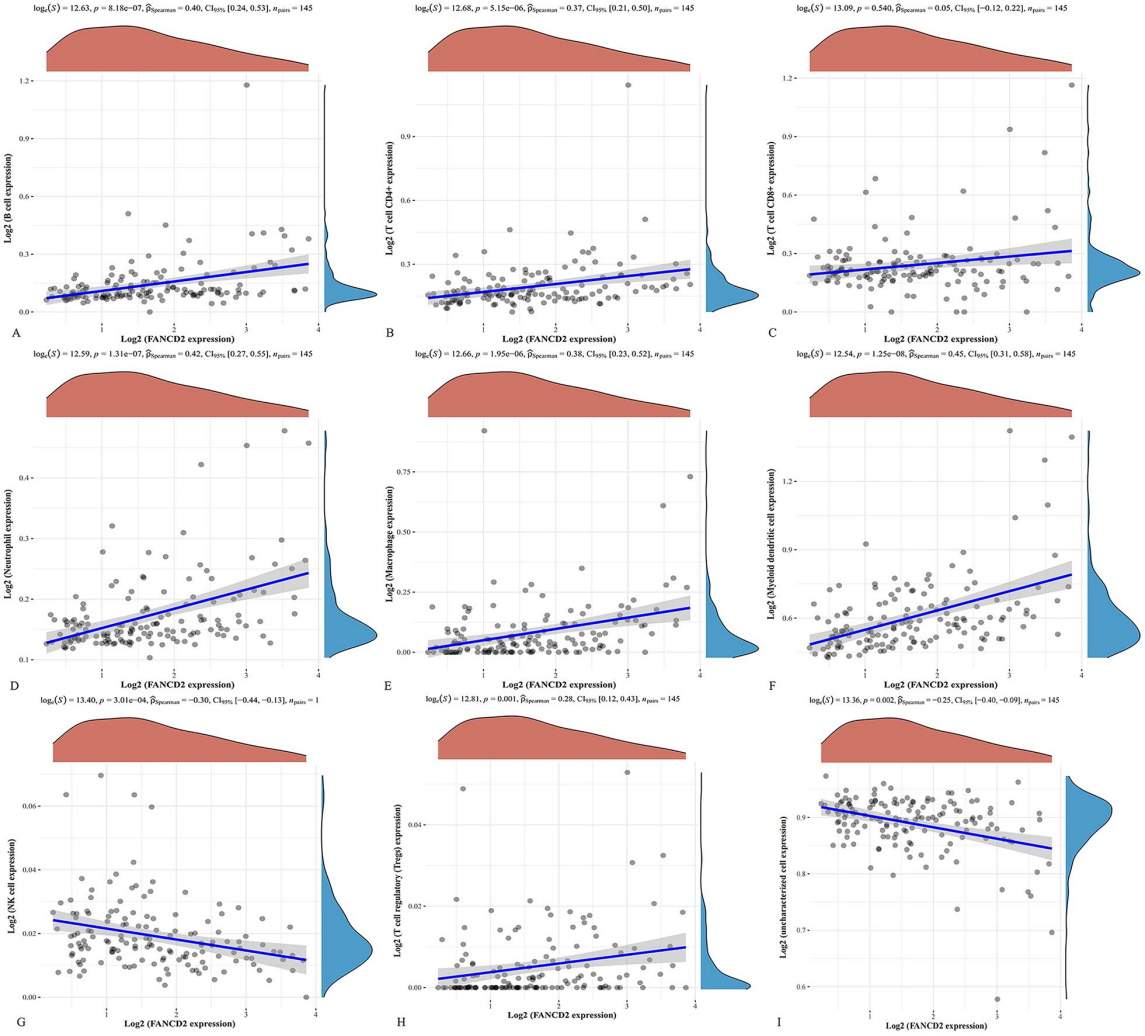
The correlation between the expression of FANCD2 and immune cells. The correlation between the expression of FANCD2 and B cells (**A**), CD8 + T cells (**B**), CD4 + T cells (**C**), neutrophils (**D**), macrophage (**E**), myeloid dendritic (**F**), NK cells (**G**), Treg cells (**H**) and uncharacterized cell (**I**)

Using the TIMER and QUANTISEQ algorithm, we found a positive correlation between the expression of FANCD2 in Hepatitis B-related HCC and the infiltration levels of B cells (P, 8.18e−07; P Spearman, 0.4), CD4 + T cells (P, 5.15e−06; P Spearman, 0.37), CD8 + T cells (P, 0.54; P Spearman, 0.05), Neutrophil (P, 1.31e−07; P Spearman, 0.42), Macrophage (P, 1.95e−06; P Spearman, 0.38), Myeloid dendritic cells (P, 1.25e−08; P Spearman, 0.45), and Treg cells (P, 0.001; P Spearman, 0.28) in Fig. 6. In contrast, we found a negative correlation between the expression of FANCD2 and infiltration levels of NK cells (*P* = 3.01e−04; P Spearman, −0.3).

**Fig. 7:**
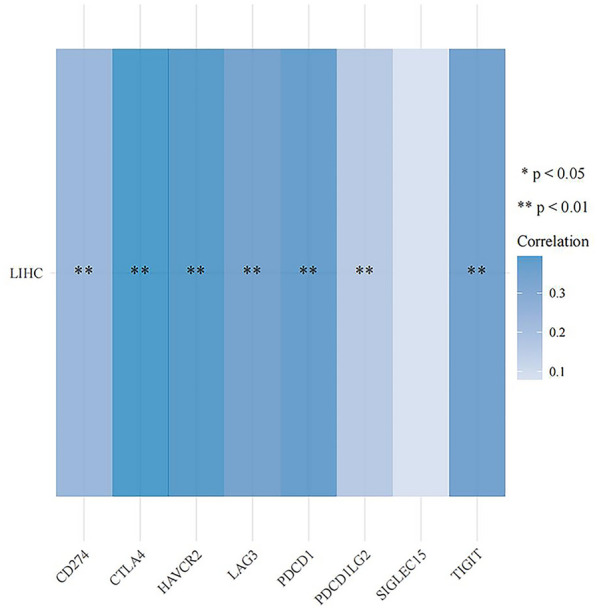
The correlations between FANCD2 and immune checkpoints gene

**Fig. 8:**
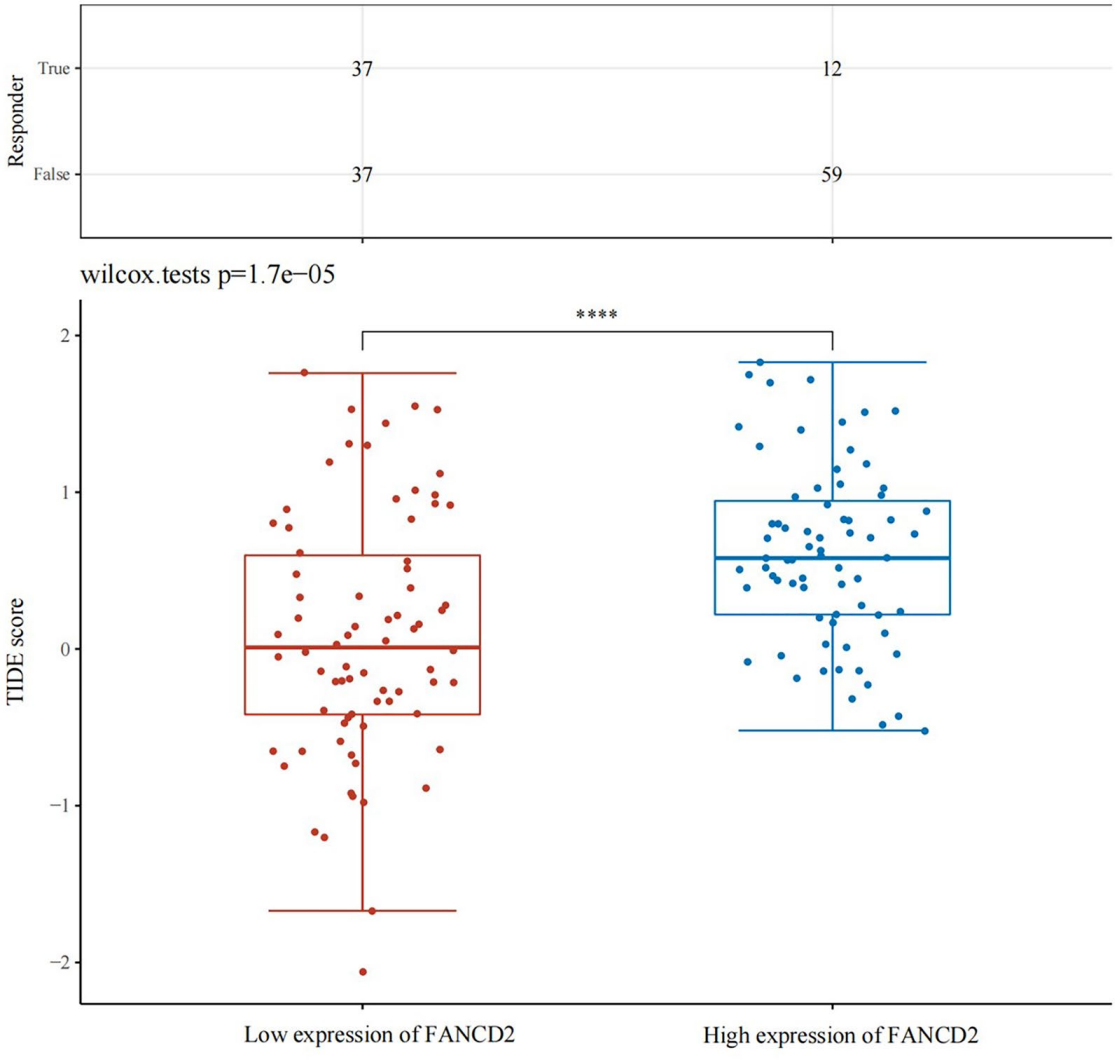
The TIDE scores in the low expression of FANCD2 and high expression of FANCD2. TIDE, tumor immune dysfunction and exclusion

The correlation between FANCD2 expression and immune checkpoints was assessed. In Hepatitis B-related HCC, the outcomes demonstrated that FANCD2 was positively correlated with the expression of immune checkpoints, including CD274 (*P* = 0.008; P Spearman, 0.22), HAVCR2 (*P* = 7.54e−07; P Spearman, 0.40), LAG3 (*P* = 5.16e−07; P Spearman, 0.41), PDCD1 (*P* = 1.16e-05; P Spearman, 0.36), and TIGIT (*P* = 1.63e−05; P Spearman, 0.35) in Fig. 7. CTLA4 and FANCD2 were also positively correlated (*P* = 5.16e−07; P Spearman, 0.41) in Hepatitis B-related hepatocellular carcinoma in Fig. 7. Figure 8 shows that highly expressed FANCD2 had a higher TIDE score.

### Protein interaction network analysis of FANCD2

**Fig. 9:**
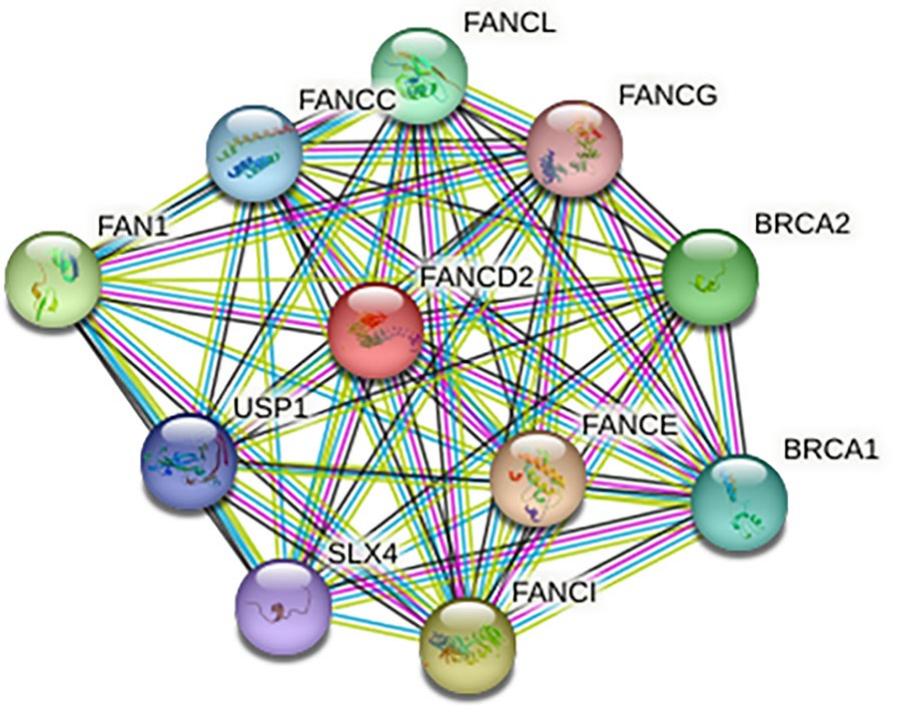
Protein Interaction Network Analysis of FANCD2

The online STRING tool revealed that FANCD2 mainly interacted with BRCA1, BRCA2, FAN1, FANCC, FANCE, FANCG, FANCI, SLX4, and USP1 (Fig. 9).

### Dryness index and TMB/MSI

**Fig. 10:**
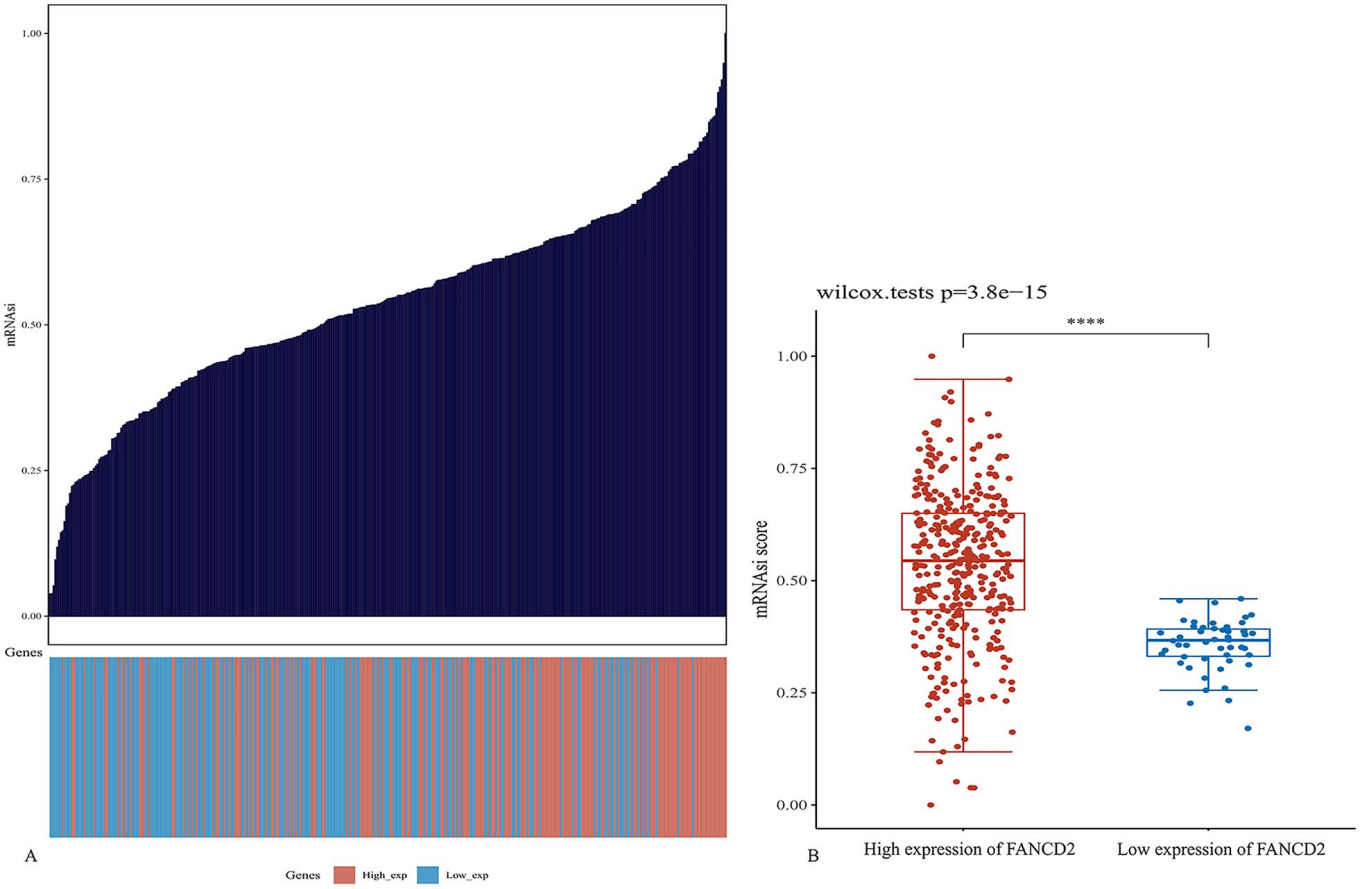
Correlation analysis of mRNAsi score and the expression of FANCD2. The top graph shows the distribution of mRNAsi score from low to high, and the bottom shows the expression of FANCD2 (**A**). The correlation analysis of mRNAsi score and the expression of FANCD2 (**B**)

**Fig. 11:**
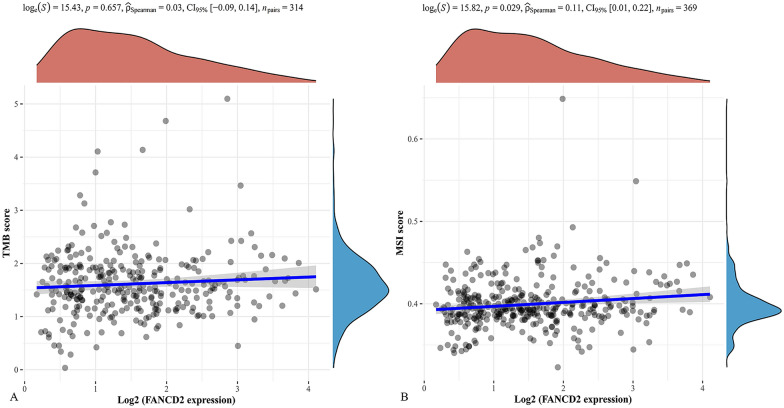
Correlation analysis between the expression of FANCD2 and tumor mutation burden (TMB) and microsatellite instability (MSI)

Figure 10 shows the correlation analysis between the mRANsi score and the expression of FANCD2. The dryness index was higher in Hepatitis B-related HCC tissues than in normal tissues (*P* = 2.6e−12). The higher the expression of FANCD2, the stronger the tumor dryness index of Hepatitis B-related HCC (*P* = 1.59e−07; *P* = 0.42; 95% CI, [0.27, 0.55]). Figure 11 shows the correlation analysis between FANCD2 and the TMB**/**MSI expression. FANCD2 was positively correlated with MSI in Hepatitis B-related HCC (*P* = 0.023 and P Spearman, 0.19). However, FANCD2 expression was not associated with TMB.

### Genes involved in iron metabolism and pathway

**Fig. 12:**
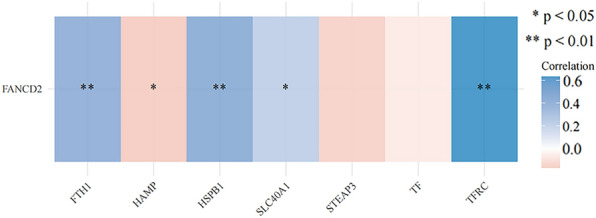
The correlations between FANCD2 and pathway

**Fig. 13:**
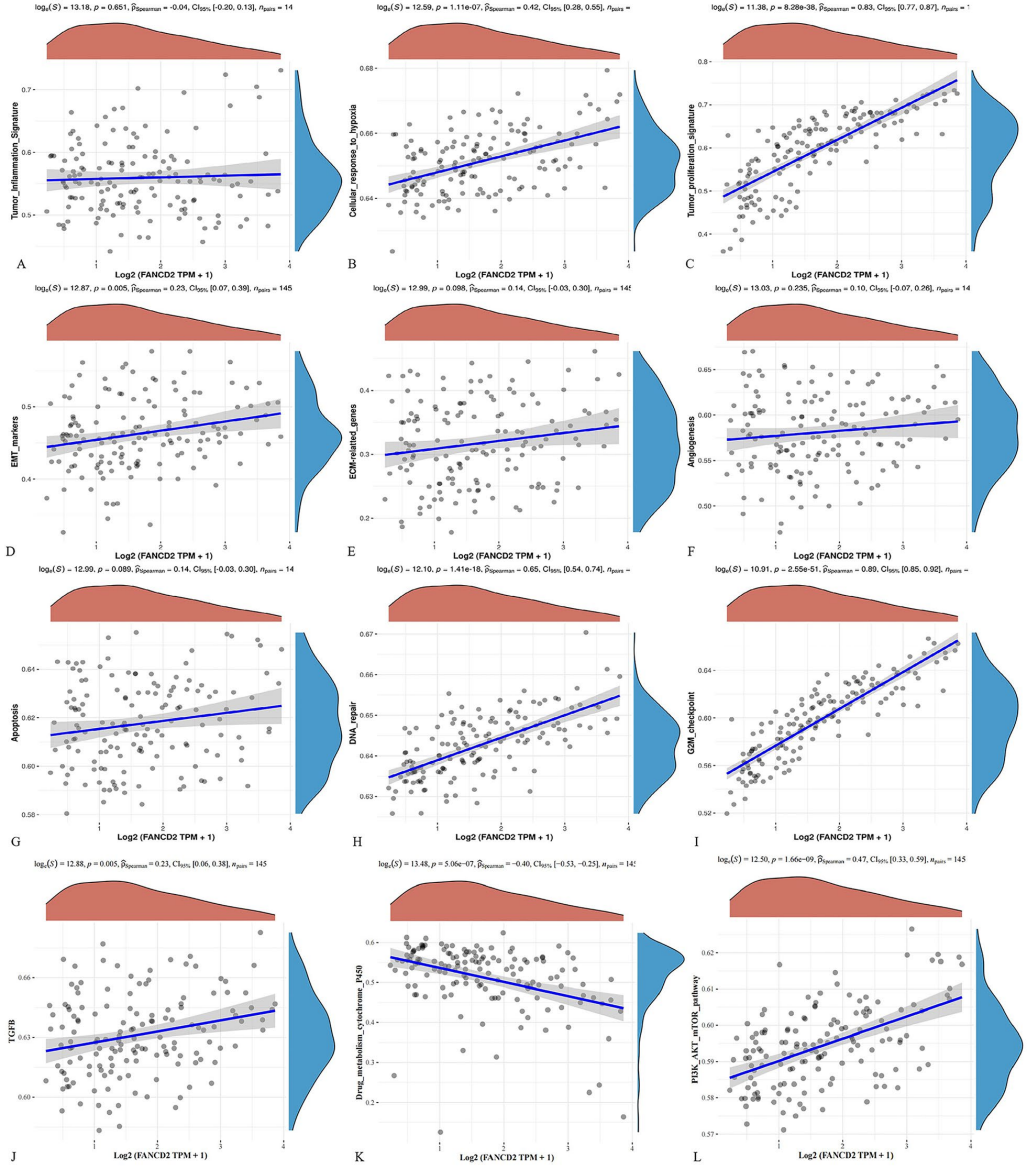
Spearman correlation analysis of IC50 score of sorafenib and the expression of FANCD2

FTH1, HAMP, HSPB1, SLC40A1, STEAP3, TF, and TFRC are the genes involved in iron metabolism. FANCD2 was positively correlated with the expression of FTH1, HSPB1, SLC40A1, and TFRC and negatively correlated with HAMP (Fig. 12). Using ssGSEA, the correlation between FANCD2 and this pathway was analyzed in Hepatitis B-related HCC (Fig. 13). The expression of FANCD2 was positively correlated with the tumor proliferation signature, DNA repair, and cellular response to hypoxia. FANCD2 expression negatively correlated with pathways such as drug metabolism cytochrome P450.

### Correlation between FANCD2 Expression and Sorafinib's IC50 score

**Fig. 14:**
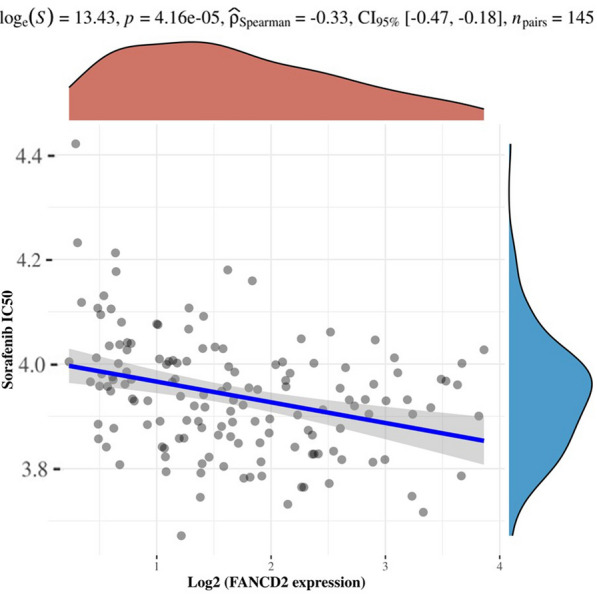
The correlations between FANCD2 and genes involved in iron metabolism

In the TCGA dataset, we determined the relationship between FANCD2 and sorafenib IC50 scores using Spearman's correlation analysis. Figure 14 shows that FANCD2 expression negatively correlated with the IC50 value of sorafenib in Hepatitis B-related HCC (P, 4.16e-05; Spearman, −0.33; CL 95%, [−0.47, −0.18]).

## Discussion

Hepatitis B-related HCC is the most common type of HCC. People infected with hepatitis B are 14 to 223 times more likely to develop HCC than those without hepatitis B infection [23]. The prognosis of different types of HCC differs after treatment [24]. Hepatitis B-related and non-viral HCC respond differently to immunotherapy [25]. Therefore, there is an urgent need to identify novel prognostic biomarkers for HB-related HCC. Ferroptosis has been found to play a crucial role in the pathogenesis of various liver diseases, such as HCC, non-alcoholic fatty liver disease, alcoholic liver disease, and drug-induced liver injury [26]. Related inducers and inhibitors of ferroptosis, such as Ferrostatin-1 and erastin, have been used to treat liver disease [27, 28]. Ferroptosis is also involved in developing Hepatitis B-related HCC [29, 30] and is associated with resistance to first-line chemotherapeutic sorafenib [31]. FANCD2 may be involved in hepatocyte injury caused by Hepatitis B [32]. Consequently, identifying additional ferroptosis-associated prognostic and diagnostic biomarkers for Hepatitis B-related HCC is crucial.

FANCD2 is a ferroptosis suppressor involved in DNA repair and has been studied in other cancers [33–36]. High FANCD2 expression in endometrial cancer correlates with a poor overall survival rate [37]. Li et al. found that silencing FANCD2 suppresses osteosarcoma cell viability, migration, invasion, and tumor growth [38]. In addition, a high expression level of FANCD2 is associated with chemotherapeutic drug resistance in glioblastomas [39]. FANCD2 is a key factor in maintaining melanoma cell proliferation and survival and is a promising target for the treatment of melanoma [36]. Abnormal FANCD2 expression has been linked to poor prognosis and more aggressive tumors [40–42]. Downregulation of FANCD2 expression significantly inhibits the proliferation of esophageal cancer cells, affecting tumor formation and metastasis, and FANCD2 is a biomarker of esophageal cancer [43]. In future basic and clinical studies, a continued focus on the role of FANCD2 in the development of tumors, metastasis, and drug resistance is critical.

In this study, we analyzed the relationship between the ferroptosis-related gene FANCD2 and the development, prognosis, treatment, immunity, and other related functions of Hepatitis B-related HCC using the TCGA and GEO databases. Our study reveals that FANCD2 is significantly upregulated in Hepatitis B-related HCC and is a promising diagnostic and prognostic biomarker. Furthermore, the expression of FANCD2 was found to increase progressively with the increase in tumor grades, with the highest expression observed in grades 3 and 4. High FANCD2 expression was associated with poor prognosis in Hepatitis B-related HCC and was shown to be an independent prognostic factor by multivariate Cox analysis. In addition, our study demonstrated that the higher the expression of FANCD2, the stronger the stem cell characteristics of tumor cells, with lower differentiation and higher proliferative capacity in Hepatitis B-related HCC. These results demonstrate that FANCD2 may serve as a biomarker for the prognosis and diagnosis of Hepatitis B-related HCC.

We found a positive correlation between FANCD2 expression and Tregs, B cells, CD4 + T cells, CD8 + T cells, neutrophils, and macrophage infiltration in Hepatitis B-related HCC. FANCD2 may influence the immune microenvironment of tumors. Tregs play a crucial role in tumor immunity by suppressing the immune response of tumors and boosting their development and progression [44, 45]. In contrast, a negative correlation was observed between FANCD2 expression and NK cell infiltration. Patients with liver cancer and low NK cell numbers have a worse prognosis [46, 47]. The expression of FANCD2 was associated with the infiltration of immune cells and the expression of immune checkpoint-related genes. Immune checkpoint blockade therapy, a critical treatment in tumor immunotherapy, has achieved good therapeutic efficacy against many tumors, such as colon cancer and melanoma [48, 49]. While immune checkpoint blockade has shown significant progress in cancer treatment, it is not universally effective for all patients [50]. Based on our study, we found that high expression of FANCD2 is associated with a higher TIDE score in Hepatitis B-related HCC. A higher TIDE score suggests a higher probability of immune escape, worse efficacy, and shorter survival with immune checkpoint-blocking therapy. These findings indicate that FANCD2 may be a potential target for immunotherapy in Hepatitis B-related HCC. The results of our study can aid in making informed clinical treatment decisions.

FANCD2 regulates the expression of genes involved in iron metabolism, such as FTH1, HAMP, HSPB1, SLC40A1, STEAP3, TF, and TFRC, to affect ferroptosis. Our study found that the expression of FANCD2 was associated with the expression of the iron metabolism genes FTH1, HAMP, HSPB1, SLC40A1, and TFRC. Therefore, FANCD2 may affect ferroptosis by altering the iron metabolism in Hepatitis B-related HCC. In addition, we used ssGSEA to analyze the correlation between FANCD2 expression and this pathway. FANCD2 is associated with multiple pathways, including DNA repair and tumor proliferation signatures, in Hepatitis B-related HCC. A protein-interaction network for FANCD2 was constructed using the STRING online tool. FANCD2 interacts primarily with BRCA1, BRCA2, FAN1, FANCC, FANCE, FANCG, FANCI, SLX4, and USP1, most of which are highly expressed in Hepatitis B-related HCC. FANCD2 and other interacting proteins are also involved in DNA damage repair. Inhibitors of DNA repair processes are highly effective in treating carcinomas [51, 52]. Thus, FANCD2 may serve as a novel therapeutic target for Hepatitis B-related HCC.

Individuals with HBV infection are at higher risk of developing liver cancer and cirrhosis. Furthermore, HBV is transmitted to people without antibodies, causing related diseases and increasing the burden on society [53]. Sorafenib, the first effective drug approved for HCC, improves the survival rate of patients [54–56]. Our study revealed a negative correlation between FANCD2 expression and the IC50 value of sorafenib in Hepatitis B-related HCC, indicating that patients with higher FANCD2 expression are more likely to have a better therapeutic response to sorafenib.

This study provides new evidence for the diagnosis, prognosis, and targeted therapy of patients with Hepatitis B-related HCC. This study had some limitations. Data were obtained from online databases and were not validated in vitro or in vivo.

## Conclusion

Our study showed that the expression of FANCD2 was increased and that high FANCD2 expression was associated with poor outcomes and unfavorable immune infiltration in Hepatitis B-related HCC. Thus, FANCD2 could be a potential diagnostic and prognostic biomarker for Hepatitis B-related HCC.

## Data Availability

The data that support the findings of this study are available from the corresponding author upon reasonable request.
